# Tripraseodymium penta­iron(III) dodeca­oxide, Pr_3_Fe_5_O_12_: a synchrotron radiation study

**DOI:** 10.1107/S1600536809038100

**Published:** 2009-10-03

**Authors:** Takashi Komori, Terutoshi Sakakura, Yasuyuki Takenaka, Kiyoaki Tanaka, Takashi Okuda

**Affiliations:** a Graduate School of Materials Science and Engineering, Nagoya Institute of Technology, Gokiso-cho, Showa-ku, Japan; b Hokkaido University of Education HAKODATE, Yahata-cho, Hakodate-shi, Japan

## Abstract

The title compound, penta­iron tripraseodymium dodeca­oxide (PrIG), has an iron garnet structure. There are two Fe site symmetries. One of the Fe atoms is coordinated by six O atoms, forming a slightly distorted octa­hedron, and has 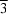 site symmetry. The other Fe atom is coordinated by four O atoms, forming a slightly distorted tetra­hedron, and has 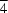 site symmetry. FeO_6_ octa­hedra and FeO_4_ tetra­hedra are linked together by corners. The Pr atom is coordinated by eight O atoms, forming a distorted dodeca­hedron, and has 222 site symmetry. The O atoms occupy the general positions.

## Related literature

The title compound is isotypic with the *Ia* 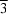 *d* form of Y_3_Fe_5_O_12_ (YIG). For related structures, see: Bonnet *et al.* (1975 ▶). For details of the crystal growth from low-temperature liquid-phase epitaxy, see: Fratello *et al.* (1986 ▶). For the extinction correction, see: Becker & Coppens (1975 ▶). X-ray intensities were measured avoiding multiple diffraction, see: Takenaka *et al.* (2008 ▶).

## Experimental

### 

#### Crystal data

Pr_3_Fe_5_O_12_
*M* *_r_* = 893.98
Cubic, 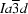

*a* = 12.6302 (3) Å
*V* = 2014.79 (8) Å^3^
*Z* = 8
Synchrotron radiation
λ = 0.67171 Å
μ = 17.41 mm^−1^
*T* = 298 K
0.035 mm (radius)

#### Data collection

Rigaku AFC four-circle diffractometer
Absorption correction: for a sphere [transmission coefficients for spheres tabulated in *International Tables C* (1992), Table 6.3.3.3, were interpolated with Lagrange’s method (four-point interpolation; Yamauchi *et al.*, 1965 ▶)] *T*_min_ = 0.413, *T*_max_ = 0.441
9351 measured reflections
1728 independent reflections
1728 reflections with *F* > 3σ(*F*)
*R*_int_ = 0.016

#### Refinement

*R*[*F* ^2^ > 2σ(*F* ^2^)] = 0.019
*wR*(*F* ^2^) = 0.021
*S* = 1.06
9351 reflections
17 parameters
Δρ_max_ = 2.52 e Å^−3^
Δρ_min_ = −3.16 e Å^−3^

### 

Data collection: *AFC-5*, specially designed for PF-BL14A (Rigaku, 1984 ▶) and *IUANGLE* (Tanaka *et al.*, 1994 ▶); cell refinement: *RSLC-3 UNICS* system (Sakurai & Kobayashi, 1979 ▶); data reduction: *RDEDIT* (Tanaka, 2008 ▶); program(s) used to solve structure: *QNTAO* (Tanaka *et al.*, 2008 ▶); program(s) used to refine structure: *QNTAO*; molecular graphics: *ATOMS for Windows* (Dowty, 2000 ▶); software used to prepare material for publication: *RDEDIT*.

## Supplementary Material

Crystal structure: contains datablocks global, I. DOI: 10.1107/S1600536809038100/br2121sup1.cif

Structure factors: contains datablocks I. DOI: 10.1107/S1600536809038100/br2121Isup2.hkl

Additional supplementary materials:  crystallographic information; 3D view; checkCIF report

## Figures and Tables

**Table:** 

Pr1—O1	2.42410 (10)
Pr1—O1^i^	2.54010 (10)
Fe1—O1	2.03220 (10)
Fe2—O1^ii^	1.87450 (10)

**Table:** 

O1—Fe1—O1^i^	85.87 (1)
O1^ii^—Fe2—O1^iii^	114.39 (1)
O1^ii^—Fe2—O1^iv^	100.02 (1)

Symmetry codes: (i) 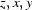; (ii) 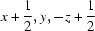; (iii) 
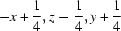
; (iv) 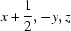.

**Fig. 1.**
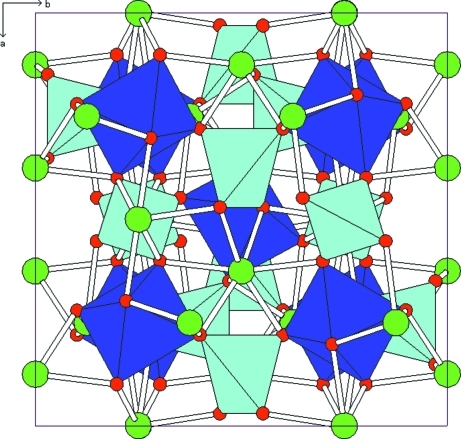
The structure of Pr3Fe5O12. Small red and large green spheres represent O and Pr atoms, respectively. Purple octahedron and blue tetrahedron represent FeO6 and FeO4 units, respectively.

**Fig. 2.**
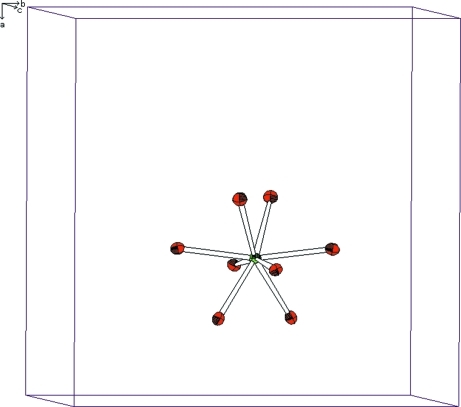
View of PrO8 with displacement ellipsoids at the 90% probability level. Green and red ellipsoids represent Pr and O atoms, in Fig.1.

## supplementary crystallographic information

### Comment

The title compound, Pr_3_Fe_5_O_12_ (PrIG), was difficult to be grown. It was grown by the low-temperature-liquid-phase epitaxy for the first time by Fratello *et al.* (1986). Though the crystal structure was assumed as iron-garnet-type structure by lattice constant and extinction rule, the complete structure was not determined. In this paper, we determine the O atom position and the complete structure by the full matrix least-squares program QNTAO. Since the R-factor is small and the residual density has no significant peaks where no atoms exists, the structure was finally determined to be iron-garnet structure. It is isotypic with the Ia3d form of Y_3_Fe_5_O_12_ (YIG). (Bonnet *et al.*, 1975). The Pr atom is coordinated by eight oxygen atoms. It forms a distorted dodecahedron. There are two Fe site symmetries. One of the Fe atom is coordinated by six oxygen atoms. It forms a slitely distorted octahedron. The other Fe atom is coordinated by four oxygen atoms. It forms a slightly distorted tetrahedron. FeO_6_ octahedron and FeO_4_ tetrahedron are linked together by corners. The structure of PrIG is drawn in Fig.1. And displacement ellipsoids of PrO_8_ is drawn in Fig.2.

### Experimental

Single crystals of praseodymium iron garnet were prepared by low temperature liquid phase epitaxy on Sm_3_(ScGa)_5_O_12_ seeds with lattice parameters near the projected values for PrIG.

### Refinement

X-ray intensities were measured avoiding multiple diffraction. (Takenaka *et al.*,  2008).

### Crystal data

**Table:**

Pr_3_Fe_5_O_12_	*D*_x_ = 5.894 Mg m^−^^3^
*M**_r_* = 893.98	Synchrotron radiation, λ = 0.67171 Å
Cubic, *I**a*3*d*	Cell parameters from 9 reflections
Hall symbol: -I 4bd 2c 3	θ = 17.5–52.3°
*a* = 12.6302 (3) Å	µ = 17.41 mm^−^^1^
*V* = 2014.79 (8) Å^3^	*T* = 298 K
*Z* = 8	Sphere, black
*F*(000) = 3224	0.04 mm (radius)

### Data collection

**Table:**

Rigaku AFC four-circle diffractometer	1728 independent reflections
Si 111	1728 reflections with *F* > 3σ(*F*)
Detector resolution: 1.25 × 1.25° pixels mm^-1^	*R*_int_ = 0.016
ω/2θ scans	θ_max_ = 68.3°, θ_min_ = 3.7°
Absorption correction: for a sphere [Transmission coefficients for spheres tabulated in International Tables C (1992), Table 6.3.3.3, were interpolated with Lagrange's method (four-point interpolation (Yamauchi *et al.*, 1965)]	*h* = −9→34
*T*_min_ = 0.413, *T*_max_ = 0.441	*k* = −9→32
9351 measured reflections	*l* = −9→34

### Refinement

**Table:**

Refinement on *F*	Primary atom site location: isomorphous structure methods
Least-squares matrix: full	Weighting scheme based on measured s.u.'s
*R*[*F*^2^ > 2σ(*F*^2^)] = 0.019	(Δ/σ)_max_ = 0.003
*wR*(*F*^2^) = 0.021	Δρ_max_ = 2.52 e Å^−^^3^
*S* = 1.06	Δρ_min_ = −3.16 e Å^−^^3^
9351 reflections	Extinction correction: B–C type 1 Gaussian isotropic (Becker & Coppens, 1975)
17 parameters	Extinction coefficient: 0.255 (5)

### Fractional atomic coordinates and isotropic or equivalent isotropic displacement parameters (Å^2^)

**Table:**

	*x*	*y*	*z*	*U*_iso_*/*U*_eq_	
Pr1	0.125000	0.000000	0.250000	0.00531 (1)	
Fe1	0.000000	0.000000	0.000000	0.00512 (1)	
Fe2	0.375000	0.000000	0.250000	0.00533 (1)	
O1	−0.029622 (2)	0.052553 (2)	0.149166 (2)	0.00711 (5)	

### Atomic displacement parameters (Å^2^)

**Table:**

	*U*^11^	*U*^22^	*U*^33^	*U*^12^	*U*^13^	*U*^23^
Pr1	0.00406 (2)	0.00594 (2)	0.00594 (2)	0	0	0.00111 (1)
Fe1	0.00512 (2)	0.00512 (2)	0.00512 (2)	−0.00023 (1)	−0.00023 (1)	−0.00023 (1)
Fe2	0.00411 (3)	0.00594 (2)	0.00594 (2)	0	0	0
O1	0.00718 (8)	0.00829 (8)	0.00587 (7)	−0.00004 (6)	0.00080 (6)	0.00038 (6)

### Geometric parameters (Å, °)

**Table:**

Pr1—O1	2.42410 (10)	Fe1—O1^i^	2.03220 (10)
Pr1—O1^i^	2.54010 (10)	Fe1—O1^viii^	2.03220 (10)
Pr1—O1^ii^	2.42410 (10)	Fe1—O1^ix^	2.03220 (10)
Pr1—O1^iii^	2.54010 (10)	Fe1—O1^x^	2.03220 (10)
Pr1—O1^iv^	2.42410 (10)	Fe1—O1^xi^	2.03220 (10)
Pr1—O1^v^	2.54010 (10)	Fe2—O1^xii^	1.87450 (10)
Pr1—O1^vi^	2.42410 (10)	Fe2—O1^iv^	1.87450 (10)
Pr1—O1^vii^	2.54010 (10)	Fe2—O1^xiii^	1.87450 (10)
Fe1—O1	2.03220 (10)	Fe2—O1^vi^	1.87450 (10)
			
O1—Pr1—O1^i^	67.75 (1)	O1—Fe1—O1^viii^	85.87 (1)
O1—Pr1—O1^ii^	72.66 (1)	O1—Fe1—O1^ix^	180.00
O1—Pr1—O1^iii^	124.91 (1)	O1—Fe1—O1^x^	94.13 (1)
O1—Pr1—O1^iv^	111.18 (1)	O1—Fe1—O1^xi^	94.13 (1)
O1—Pr1—O1^v^	73.25 (1)	O1^xii^—Fe2—O1^vi^	114.39 (1)
O1—Pr1—O1^vi^	159.51 (1)	O1^xii^—Fe2—O1^iv^	114.39 (1)
O1—Pr1—O1^vii^	95.43 (1)	O1^xii^—Fe2—O1^xiii^	100.02 (1)
O1—Fe1—O1^i^	85.87 (1)		

Symmetry codes:  (i) *z*, *x*, *y*; (ii) *x*, −*y*, −*z*+1/2; (iii) *z*, −*x*, −*y*+1/2; (iv) −*x*+1/4, −*z*+1/4, −*y*+1/4; (v) −*z*+1/4, −*y*+1/4, −*x*+1/4; (vi) −*x*+1/4, *z*−1/4, *y*+1/4; (vii) −*z*+1/4, *y*−1/4, *x*+1/4; (viii) *y*, *z*, *x*; (ix) −*x*, −*y*, −*z*; (x) −*z*, −*x*, −*y*; (xi) −*y*, −*z*, −*x*; (xii) *x*+1/2, *y*, −*z*+1/2; (xiii) *x*+1/2, −*y*, *z*.
